# Isometric single-joint rate of force development shows trivial to small associations with jumping rate of force development, jump height, and propulsive duration

**DOI:** 10.1016/j.jsampl.2022.100006

**Published:** 2022-10-22

**Authors:** Bas Van Hooren, Žiga Kozinc, Darjan Smajla, Nejc Šarabon

**Affiliations:** a NUTRIM School of Nutrition and Translational Research in Metabolism, Maastricht University Medical Centre+, Department of Nutrition and Movement Sciences, Maastricht, the Netherlands; b University of Primorska, Faculty of Health Sciences, Izola, Slovenia; c University of Primorska, Andrej Marušič Institute, Koper, Slovenia; d InnoRenew CoE, Human Health Department, Izola, Slovenia; e S2P,Science to Practice, Ltd., Laboratory for Motor Control and Motor Behavior, Ljubljana, Slovenia

**Keywords:** Countermovement jump, Squat jump, Correlation, Transfer, Unilateral, Linear regression

## Abstract

**Objectives:**

The association between single-joint isometric rate of force development (RFD_ISO_) and jumping outcomes remain largely unexplored. Further, the importance of RFD assessed during jumping for jump height and duration (i.e. time from jump onset to take-off) remains ambiguous. We therefore investigated these associations in a large heterogenous sample.

**Design:**

Cross-sectional study.

**Methods:**

Three-hundred-twenty-six male and female basketball and tennis players, and physical education students performed the bilateral squat jump (SJ) and both bilateral and unilateral countermovement jumps (CMJ). Single-joint RFD_ISO_ was assessed for the hip extensors, knee extensors and ankle extensors and associations between relevant outcomes were computed.

**Results:**

Knee and hip extensors RFD_ISO_ showed small positive correlations with RFD_SJ_ and RFD_CMJ_. Ankle extensors RFD_ISO_ showed a moderate positive correlation with RFD_SJ_ and RFD_CMJ_. RFD_ISO_ showed small to moderate correlations with CMJ and SJ jump height, but trivial correlations with jump duration. Stepwise linear regression showed that a combination of RFD_ISO_ from different muscle groups explained a small to moderate variance in jump height (∼23–28%), duration (∼2–3%), and RFD during jumping (∼19–28%). RFD_SJ_ showed small positive and moderate negative correlations with SJ height and duration, respectively while these correlations were small and trivial for the CMJ.

**Conclusions:**

The positive correlations between RFD during jumping and jump height, and negative correlation with jump duration imply that improving RFD during jumping could benefit jump performance. However, the mostly small correlations between single-joint RFD_ISO_ and jumping RFD suggests that single-joint RFD_ISO_ assessments provide only limited information regarding the RFD in sports-related movements.

## Highlights

• Dynamic rate of force development during jumping shows a small positive association with jump height and trivial to moderate negative correlations with jump duration.
• Single-joint rate of force development shows mostly small correlations with jumping rate of force development.
• Single-joint rate of force development shows small to moderate associations with jump height and trivial associations with jump duration
• Stepwise linear regression showed that a combination of RFD_ISO_ from different muscle groups explained a small to moderate variance in jump height (∼23–28%), duration (∼2–3%), and RFD during jumping (∼19–28%).

## 1 Introduction

Successful sports performance often requires large force production during a short time period and a high rate of force development (RFD) is therefore considered an important performance determining factor [1]. A higher RFD can theoretically improve performance by a) increasing the net impulse (e.g., larger jump height [2] or greater acceleration during the ground contact of running), b) shortening the time needed to perform the movement (e.g., shorter jump duration with the same height), or c) a combination of both. Importantly, however, RFD may also increase while the net impulse (and thus jump height) decreases in situations where the jump duration decreases but peak force remains similar. Although a higher RFD during the movement may contribute to performance, RFD metrics have been shown to exhibit relatively low reliability during dynamic movements such as jumping [[3], [4], [5]]. RFD is therefore often assessed in single- and multi-joint isometric or isokinetic conditions to ensure high reliability, and in the case of single-joint assessments also to isolate the performance of one muscle group, which in turn may better inform on which muscle groups to train to improve overall RFD. While correlations between multi-joint isometric (e.g., mid-thigh pull or squat) RFD, and RFD during vertical jumping vary widely and are often trivial to small [[6], [7], [8], [9]], the relation between single-joint isometric RFD (RFD_ISO_) and RFD during jumping has not been investigated. It remains therefore unknown if single-joint assessments are useful to predict jumping RFD either when considered in isolation or when combined into a multiple linear regression model to investigate their combined influence.

The relevance of single-joint RFD to more holistic outcomes such as jump height or jump duration also remains ambiguous. Indeed some authors report significant and moderate to large associations between (normalized) single-joint [[10], [11], [12]] or multi-joint [13,14] RFD_ISO_ and jump height. Yet other authors report no significant or trivial associations between both single-joint [12,15,16] or multi-joint [7,[17], [18], [19]] RFD_ISO_ and jump height, or report conflicting findings depending on the parameter investigated [12,[20], [21], [22]]. The relatively small sample sizes in these studies (typically 12–30 participants), and methodological differences in for example the RFD calculations (e.g. mean or peak RFD, moving average window duration, and method of force production onset [[23], [24], [25]]) or testing methods (e.g., instructions, RFD from best trial vs average of a specific number of trials, duration of the test protocol [[26], [27], [28]]) may have contributed to the between-study variability in the reported correlations and lack of significant associations between RFD_ISO_ and jumping outcomes reported in some studies. A study with a large heterogenous sample would partly overcome these limitations and could also allow researchers to combine several outcomes (e.g. RFD_ISO_ from different joints) into a statistical model to investigate their combined contribution to another outcome such as jump height. However, only a small number of researchers have used multiple linear regression to investigate the contribution of multiple parameters to a specific outcome during jumping [15,29], likely because the sample sizes are often too small since at least 10 participants are recommended to be included for each predictor in a multiple linear regression analysis [30].

The primary aim of this study is therefore to investigate the association between single-joint RFD_ISO_ of the ankle, knee and hip extensors and multi-joint bilateral and unilateral dynamic RFD in vertical jumping, as well as the association between single-joint RFD_ISO_ and jump height and jump duration using both correlations and multiple linear regression in a large sample (*n* ​= ​326) of athletes from a variety of sports. Based on the findings of previously discussed studies, we hypothesized that single-joint RFD_ISO_ would only show small associations with jump height, jump duration or RFD in jumping when each joint was considered in isolation using correlation analysis. Additionally, we hypothesized that a linear regression with multiple single-joint RFD_ISO_ metrics as predictors would be able to predict at least a moderate magnitude (>20%) of jump height, jump duration and RFD during jumping.

Finally, the literature reports conflicting findings regarding the associations between RFD assessed *during* jumping and jump performance. For example, some researchers report a positive and moderate to large correlation between a higher RFD in jumping and a greater jump height [3,4,10,31], or between a higher RFD in jumping and both higher jump height and shorter jump duration [31], while other researchers report only a significant correlation between RFD and jump duration, but not jump height [32]. Yet other authors do not report any significant or trivial correlations, or report conflicting correlations between RFD in jumping and jump height or jump duration depending on the parameters investigated [29,[33], [34], [35], [36]]. These findings may partly reflect the complex association between RFD and net impulse as discussed previously. A secondary aim was therefore to investigate the associations between RFD during vertical jumping and both jump height and jump duration. We hypothesized that RFD during jumping showed a small positive association with jump height and small negative association with jump duration.

## 2 Methods

This was an observational cross-sectional study that used data collected previously for a project that investigated inter-limb asymmetries in different athletic populations. Before testing, a 20-min warm-up was performed, which consisted of 10 ​min of low-intensity jogging, 10 ​min of dynamic stretching exercises, and bodyweight resistance exercises (e.g., bodyweight squats, lunges, heel raises, side lunges). Then, individual test sections (i.e., single-joint strength, vertical jumps and assessments not related to this study) were performed in a randomized order.

The study sample comprised of male and female basketball and tennis players, and physical education students. The details regarding sample demographics are presented in Table 1. The inclusion criterion was an absence of musculoskeletal injuries in 6 months prior to testing. The participants were informed about the testing procedures and signed informed consent before participation. The experiment was approved by the Republic of Slovenia National Medical Ethics Committee (approval no. 0120–99/2018/5). An *A priori* sample size determination with an online research tool (Samplesize.net) showed that a sample size of 326 individuals allowed us to detect a correlation of *r* ​= ​0.16 with a 0.05 type I and 0.20 type II error rate, respectively. Correlations of this magnitude have previously been reported by several authors when investigating the associations between RFD and jumping outcomes [3,4,[6], [7], [8], [9], [10],^31^]. The participants were familiar with jumping tasks, as they participated in regular testing (basketball and tennis players) or performed the task as a part of their practical lessons at the faculty.

**Table 1:** Mean ​± ​SD study sample characteristics.

Group	Males/females	Age (years)	Height (cm)	Mass (kg)
Basketball (n ​= ​164)	106/58	16.8 ​± ​1.4	184.0 ​± ​9.8	76.8 ​± ​13.0
Tennis (n ​= ​104)	61/43	15.9 ​± ​3.4	172.1 ​± ​10.9	62.9 ​± ​12.7
Physical education students (n ​= ​58)	31/27	19.7 ​± ​0.6	175.1 ​± ​9.6	68.9 ​± ​11.5
Total (n ​= ​326)	198/128	17.0 ​± ​2.5	178.6 ​± ​11.5	71.0 ​± ​14.1

All participants performed bilateral and unilateral countermovement jumps (CMJ) and squat jumps (SJ) on a force plate (Kistler, model 9260AA6, Winterthur, Switzerland), as described previously [22]. Briefly, jumps were performed in a randomized order and the maximum height of two jumps was averaged and used for analysis. Rest between jumps was 1 ​min between trials and 3 ​min between CMJ and SJ. Hands were kept on the hips in both jumps and participants were instructed to jump as high and fast as possible and use a fast countermovement (CM) in the CMJ. For unilateral CMJ's, jumps with a swing of the non-support leg were excluded. The depth of the CM and start of the SJ were set at 90° knee angle. Before each jump (bilateral and unilateral), participants were asked to squat slowly under the examiner guidance, until the desired position (90° knee angle) was reached. The examiner also monitored the execution visually, to verify that the appropriate depth was reached. After stabilizing for 3 ​s [37], the SJ jump was performed without CM from a 90° knee angle. The examiner immediately inspected the force–time curve, and the jump was repeated if a CM was present (evident as decrease of force for ​∼ ​10 ​N prior to onset of force rise). Before each jump (SJ and CMJ), participants were required to squat in a controlled manner until the desired position was reached, in order to become familiarized with 90° knee angle position.

**Fig. 1:**
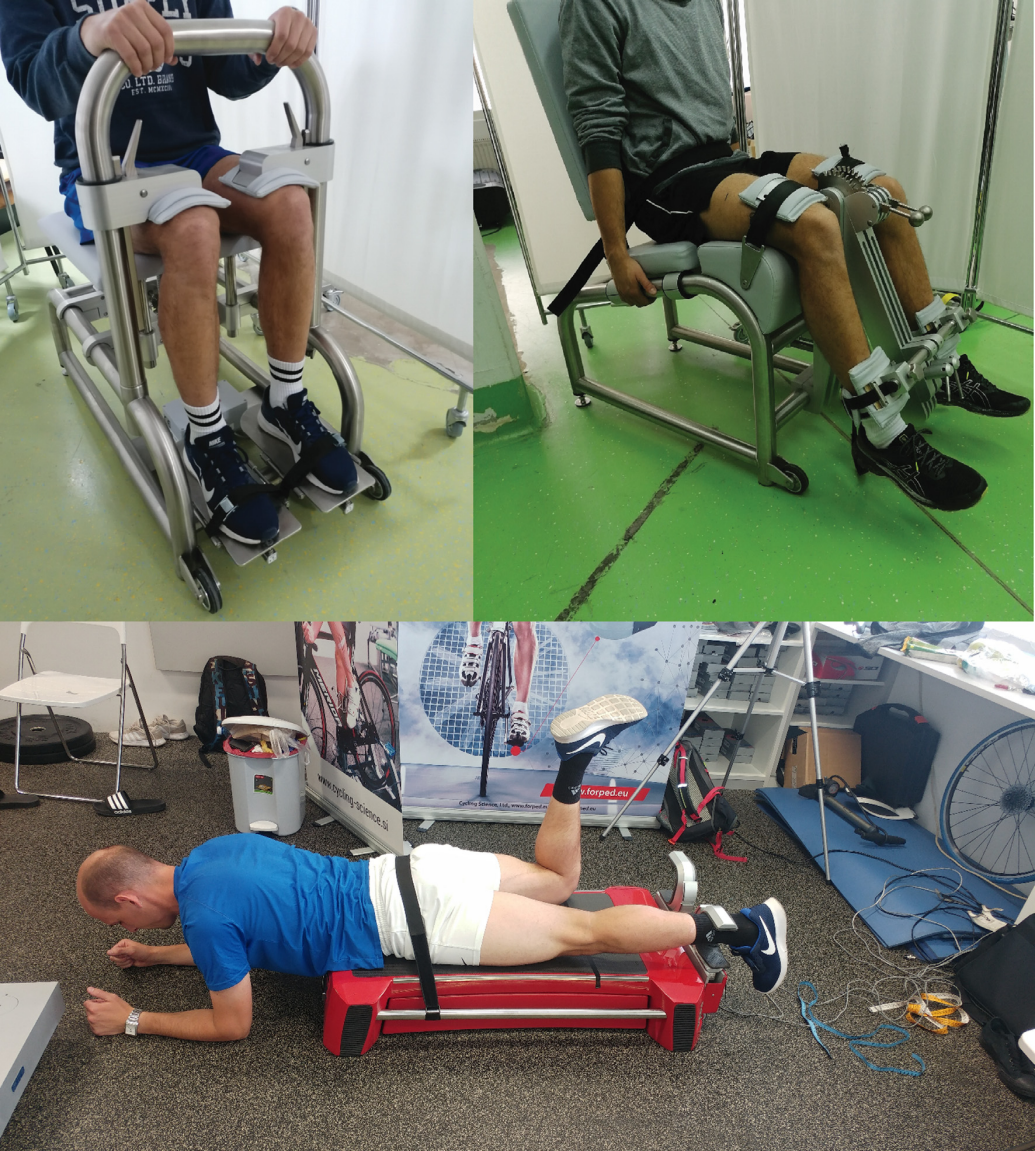
Positions for the single-joint rate of force development assessments. Left top: ankle extensors; right top: knee extensors; bottom: hip extensors.

Isometric strength assessments were performed using dynamometers with embedded force sensors as described previously [22]. Briefly, hip extension strength was assessed on a MuscleBoard dynamometer with the participants prone and hands supported on the floor with the knee in full extension and hip in neutral (anatomical) position (Fig. 1). Two “U”-shaped and padded aluminum braces, attached to uniaxial load cells (FL34-100 ​kg; Forsentek Co., Shenzhen, China), were used to measure forces (N), separately for each limb. Ankle extension strength was assessed using an isometric ankle dynamometer (S2P, Ljubljana, Slovenia). The participants were seated and tightly secured with the ankle in neutral (anatomical) position, while the knee and the hip were bent to 90°. For knee extension assessment, the participants were seated into the dynamometer (S2P, Ljubljana, Slovenia), with the hip at 90° and the knee at 60°. Fixations were provided at the waist and distal thighs. The axis of the dynamometer was aligned with the lateral femoral condyle. Participants were instructed to push as fast and hard as possible. Data was sampled at 1000 ​Hz for the knee and the ankle, and at 450 ​Hz for the hip. The difference in sampling rate was due to different inherent maximal sampling rates of the sensors. For all tasks, the participants first performed 4 familiarization trials at 50, 75, 90 and 100% of previewed maximal effort. Then, three repetitions were performed and recorded, with 1 ​min breaks. Feedback on the signals was provided online at all times. The two repetitions with the largest RFD were averaged in line with previous suggestions [23] and used for further analyses.

Hip forces were converted offline to torque using the measured lever arm (the distance from greater femoral trochanter to the point of the contact with the dynamometer), while the ankle and knee dynamometers directly measured torque production. The onset of torque rise was determined by offline analysis. In the software, the signal was shown and a marker was available, which was moved manually to the onset of contraction. After applying a moving average filter with a 5 ​ms window, peak rate of torque development (RTD) was calculated as the maximum slope of the first derivative of torque with respect to time (Δ torque/Δ time). In the remaining paper we will refer to the RTD when discussing the individual joints, and RFD_ISO_ when referred to the group of isometric RFD assessments. This is justified since the lever arms did not change during the isometric assessments.

Jumping RFD was defined as the maximum slope of the force curve during the braking phase of the CMJ (RFD_CMJ_) and the propulsive phase in the SJ (RFD_SJ_), respectively. The braking phase of the CMJ refers to the period when the vertical ground reaction force is rising above body weight until peak force [38] and the RFD during this phase has been suggested as one of the critical factors for jumping performance [39]. Although the net impulse (and thus RFD) during the braking phase will depend on the net impulse during the unweighting phase, and therefore not directly contribute to jump performance (but rather to braking of the center of mass) [38], a higher RFD during the braking phase reflects a more rapid deceleration of the center of mass and this may lead to less dissipation of elastic energy into heat [37], which in turn contributes to an enhanced propulsive impulse and thereby providing a potential mechanistic link between brake phase RFD and (propulsive) jump performance. As indirect support, a recent study reported that CMJ braking phase RFD was not significantly different from SJ RFD or RFD during an isometric leg press in the first 50 ​ms [40], suggesting a conceptual link between the RFD during jumping and isometric tasks. Peak RFD was used as an indicator of rapid force development ability as it has been shown to have a stronger association with jump height than for example average RFD [3]. Moreover, peak RFD had comparable reliability to RFD over specific time periods for the single-join assessments (online Supplementary file 1).

Jump height was calculated based on take-off velocity (V_take-off_) using (V_take-off_) [2]/(2 ​g). Jump duration was calculated as the time between jump onset and take-off. Jump onset was determined at the instant when the force curve was lowered for 3 standard deviations relative to the baseline ground reaction force for the CMJ, and at the instant when the force curve was increased for 3 standard deviations relative to the baseline ground reaction force in the SJ. Take-off was determined as the first force value lower than 10 ​N. These thresholds were used as they showed an excellent ability to detect the events of interest as compared to visual detection in pilot analysis. The standard deviation of the ground reaction force was determined during a separate 2 ​s measurement prior to CMJ and SJ initiation, while the participant stood as still as possible in line with previous guidelines [38]. Threshold based on standard deviations was used for the jump onset as it takes into account the inherent signal noise unlike the use of absolute thresholds (e.g., 10 ​N below body weight). The force traces were also manually checked for potential errors and corrected if needed.

Statistical analyses were done with SPSS (version 25.0, SPSS Inc., Chicago, USA). Descriptive statistics are reported as mean ​± ​standard deviation (SD) for both males and females separately, and combined. Normality of the raw data distribution was verified visually using histograms and Q–Q plots. Correlations among outcome variables were assessed with Pearson's correlation coefficients and interpreted as trivial (<0.1), small (0.1–0.39), moderate (0.4–0.69), strong (0.7–0.89) and very strong (>0.9). Cook's distance was computed for all correlations and outcomes from participants with a Cook's distance of >1 were considered influential outliers and excluded from analysis. However, this procedure did not result in the exclusion of any participant. Multiple linear stepwise regressions were done with either jump height, jump overall (for CMJ) and propulsive duration or jumping RFD as a dependent variable and all RFD_ISO_ variables as predictors. Predictors were included only when their contribution was significant at an alpha level of ​< ​0.05. Durbin–Watson statistics and collinearity tests were also performed. We conservatively set the thresholds for presence of collinearity at ≥3 for variance inflation factor. Additionally, a visual inspection of a scatterplot of residuals was done to confirm homoscedasticity of the residuals. For all analyses, the threshold for statistical significance was set at *p* ​< ​.05. Reliability for the assessed outcomes was determined using a using a mean rating two-way random model intraclass correlation coefficient (ICC) for consistency and standard error of measurement in absolute and percentage units. The ICC was considered <0.69 poor; 0.7–0.79; acceptable; 0.8–0.89, good; and 0.9–0.99, excellent [41].

## 3 Results

Prior to data analysis, data from three bilateral CMJs and one SJ were removed as the jump height (∼10 ​cm) was considerably lower than all other individuals and this was therefore not considered representative of a maximum effort. Supplementary file 1 reports the intraclass correlation coefficient for the assessed outcomes. Briefly, reliability ranged from poor to excellent, depending on the outcome. Jump height for example typically demonstrated excellent reliability, jump duration acceptable reliability and jumping as well as single-joint RFD showed poor reliability.

**Fig. 2:**
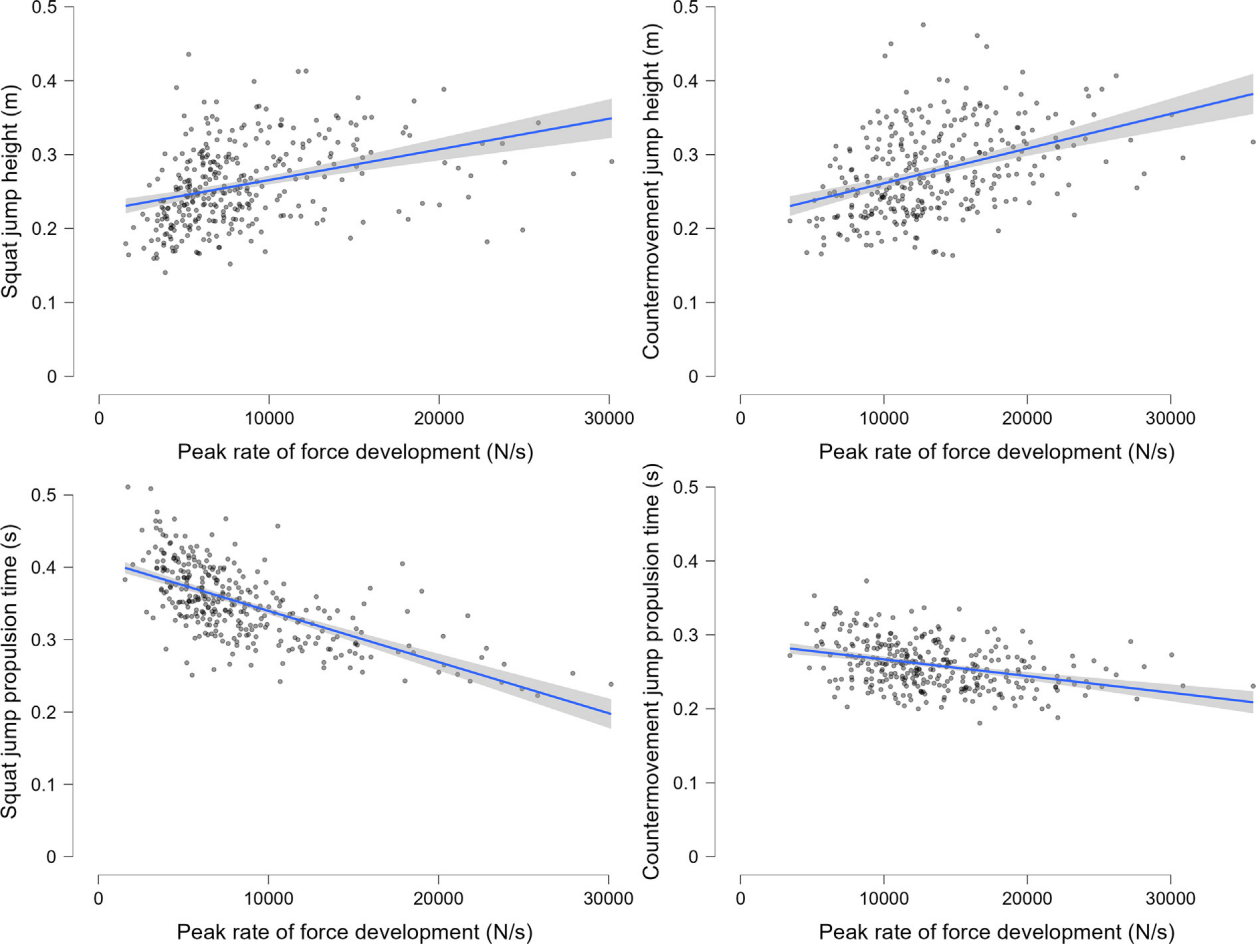
Scatterplots depicting the relationship between peak rate of force development and squat and countermovement jump height (upper panel) and duration (lower panel). Dots represent individual data points, the blue solid line represents the linear regression line, and the shaded grey band represents the 95% confidence intervals.

Table 2 reports the means and SD for all investigated variables for both males and females combined, while Table SI and SII report results separately for males and females, respectively. Table SIII reports the correlations between single-joint RFD_ISO_ and RFD_SJ_, the correlations between RFD_SJ_ and SJ height and duration, as well as the correlation between single-joint RFD_ISO_ and SJ height and SJ propulsive duration (see also Fig. 2) for both sexes. Briefly, RFD_SJ_ showed a small positive and moderate negative correlation with SJ height and SJ duration, respectively. Single-joint RFD_ISO_ metrics show mostly small correlations with SJ height and trivial correlations with SJ propulsive duration. Similarly, correlations were mostly small between single-joint RFD_ISO_ metrics and RFD_SJ_.

**Table 2:** Mean ​± ​SD for all assessed variables.

Outcome	Mean ​± ​SD bilateral outcomes	Mean ​± ​SD left leg outcomes	Mean ​± ​SD right leg outcomes
**Squat jump**			
Jump height [m]	0.26 ​± ​0.06	n.a.	n.a.
Propulsion time [s]	0.35 ​± ​0.06	n.a.	n.a.
Time to maximum force [s]	0.24 ​± ​0.07	n.a.	n.a.
Peak RFD [N/s]	8410 ​± ​4734	n.a.	n.a.
**Countermovement jump**			
Jump height [m]	0.28 ​± ​0.06	0.13 ​± ​0.04	0.14 ​± ​0.04
Jump time [s]	0.71 ​± ​0.08	0.76 ​± ​0.109	0.77 ​± ​0.109
Countermovement time [s]	0.46 ​± ​0.05	0.46 ​± ​0.06	0.46 ​± ​0.06
Propulsion time [s]	0.26 ​± ​0.03	0.30 ​± ​0.04	0.31 ​± ​0.04
Braking time [s]	0.28 ​± ​0.04	0.28 ​± ​0.05	0.29 ​± ​0.05
Time to maximum force [s]	0.49 ​± ​0.109	0.55 ​± ​0.12	0.57 ​± ​0.12
Peak RFD [N/s]	13,416 ​± ​5067	7515 ​± ​4797	8966 ​± ​4148
**Isometric Strength**			
Knee extensors peak RTD [Nm/s]	n.a.	934 ​± ​410	911 ​± ​401
Ankle extensors peak RTD [Nm/s]	1435 ​± ​530	n.a.	n.a.
Hip extensors peak RTD [Nm/s]	n.a.	998 ​± ​520	1061 ​± ​528

RFD ​= ​rate of force development; RTD ​= ​rate of torque development.

Table SVI reports similar metrics as Table SIII, but for the CMJ for both sexes. Overall, RFD_CMJ_ showed a small positive and trivial negative correlation with CMJ height and CMJ duration, respectively. Single-joint RFD_ISO_ showed small to moderate correlations with CMJ height, but trivial correlations with overall jump duration or propulsive duration. Correlations between single-joint RFD_ISO_ and RFD_CMJ_ were also mostly small. Finally, Tables SIX and SX in Supplementary file 2 report similar metrics as Table SI and SII, but for the unilateral CMJ's.

Table 3 summarizes the results of the stepwise linear regressions, with the final models being shown for the bilateral SJ and CMJ. The Durbin–Watson statistic values showed no indication of correlations between subject numbers and the magnitude of the residuals, and the plots of predicted vs standardized residuals also shows no clear violation of the homogeneity of variance assumption. The SJ height model included two predictor variables (ankle extensors RTD and right knee extensors RTD). These variables accounted for 22.6% of the variance in SJ height, of which the most (20.6%) was explained by ankle extensors RTD. The SJ duration model included one predictor (left hip extensors RTD), which accounted for only 2.5% of the variance. The RFD_SJ_ model also included one predictor (ankle extensors RTD), which explained 18.8% of the variance.

**Table 3:** Final stepwise regression models for the bilateral SJ and CMJ.

Outcome & predictors	Regression outcomes				Statistical significance	
SJ height (cm)	Adjusted R^2^	B	Std. Error	Standardized Coefficient	*t*	Sig.
Ankle extensors RTD	0.206	3.85E-5	0.001	0.365	6.234	<0.001
Left knee extensors RTD	0.226	2.38E-5	0.001	0.178	3.036	0.003
**SJ propulsion duration (ms)**						
Left hip extensors RTD	0.025	−1.70E-5	<0.001	−0.169	−3.003	0.003
**SJ RFD (N/s)**						
Ankle extensors RTD	0.188	4.005	0.470	0.437	8.521	<0.001
**CMJ height (cm)**						
Ankle extensors RTD	0.255	4.60E-5	<0.001	0.404	7.138	<0.001
Left knee extensors RTD	0.282	2.89E-5	<0.001	0.200	3.527	<0.001
**CMJ overall duration (ms)**						
No variables fit the criteria	–	–	–	–	–	–
**CMJ propulsion duration (ms)**						
Ankle extensors RTD	0.019	−9.14E-6	<0.001	−0.148	−2.620	0.009
**CMJ RFD (N/s)**						
Ankle extensors RTD	0.226	2.649	0.614	0.275	4.315	<0.001
Right knee extensors RTD	0.267	2.767	0.727	0.222	3.808	<0.001
Right hip extensors RTD	0.280	1.447	0.570	0.151	2.537	0.012

The bilateral CMJ height model included two predictor variables (ankle extensors RTD, and left knee extensors RTD). These variables accounted for 28.2% of the variance in CMJ height. No predictors met the criteria for the bilateral CMJ overall duration model. The model for bilateral CMJ propulsion phase duration included one predictor variable (ankle extensors RTD), which explained only 1.9% of the variance. The CMJ RFD model included three predictor variables (ankle extensors, and right knee and hip extensors RTD), which accounted for 28% of the variance, of which most (22.6%) was explained by the ankle extensors.

## 4 Discussion

The primary findings of this study are that single-joint RFD_ISO_ for the knee and hip extensors showed only small positive correlations with dynamic multi-joint RFD in the bilateral SJ and CMJ, and in the unilateral CMJ. Ankle extensors RTD however showed a moderate positive correlation with RFD during the bilateral SJ and CMJ, but only a small correlation with unilateral CMJ RFD. Single-joint RFD_ISO_ also showed small to moderate correlations with CMJ and SJ jump height, but trivial correlations with jump duration. In line with this, the regression model showed that a combination of RFD_ISO_ from different muscle groups generally explained a small to moderate variance in jump height (∼23–28%), duration (∼2–3%) or jumping RFD (∼19–28%). These findings therefore partly support our hypotheses regarding a) mostly small associations between jumping RFD and jump performance, and generally small correlations between single-joint RFD_ISO_ and jump height, jump (propulsive) duration or jumping RFD, and b) that a combination of multiple metrics in a linear regression was able to explain a moderate magnitude of variance for some outcomes. With regard to our second aim, RFD measured during the SJ showed a small positive and moderate negative correlation with SJ height and duration of the propulsive jump phase, respectively while these correlations were small and trivial for the CMJ, respectively.

Our findings indicate that RFD during the SJ and CMJ has small positive correlations with jump height, and moderate and trivial negative correlations with SJ and CMJ duration, respectively (Fig. 2). These findings are in agreement with previous studies that report (small) positive correlations between a higher jumping RFD and greater jump height [3,4,10,31], or between a higher jumping RFD and both higher jump height and shorter jump duration [31]. Yet, the magnitude of the correlation between RFD and jump height was small, which could explain why some studies with mostly small sample sizes have shown conflicting findings for these associations [29,[33], [34], [35], [36]]. The trivial correlation between CMJ_RFD_ and CMJ jump duration (from initiation to toe-off, also often referred to as time to takeoff) could be due to the speed of the downward phase, whereby a relatively slow downward phase and thus long CMJ duration could still be accompanied by a fast RFD. Indeed, CMJ duration and time to peak force were relatively long (Table 2) compared to other studies [3], despite instructions to perform a fast countermovement. In contrast, during the SJ there is no downward phase and a higher RFD therefore shows a more direct association with a shorter jump duration. Indeed, SJ time to peak force was considerably shorter (∼270 ​ms), which is more in line with previous studies [3].

Single-joint RFD_ISO_ showed small to moderate correlations with peak RFD_SJ_ and RFD_CMJ_, but only trivial correlations with SJ and CMJ time to peak force. Ankle extensors RTD was the only parameter that showed a moderate correlation with RFD_SJ_ and RFD_CMJ_. To the authors' knowledge, this is the first study that reports on the relationship between single-joint RFD_ISO_ and jumping RFD. In a study investigating the RFD scaling factor (i.e., the slope of the linear relationship between peak force RFD across rapid contractions of varying submaximal intensities), small correlations (r ​= ​0.27–0.31) were reported between drop jump and single-joint isometric plantarflexion tasks [33], which is in line with our findings. Similarly, the correlations between multi-joint isometric (e.g., mid-thigh pull or squat) RFD and RFD during vertical jumping are often trivial to small [[6], [7], [8]]. The small correlations between RFD_ISO_ and jumping RFD suggests that different mechanisms limit RFD_ISO_ and jumping RFD. Specifically, early (<75 ​ms) RFD in isometric or isokinetic conditions depends primarily on the rate by which muscle activation can be increased [23]. During jumping, the rate by which muscle activation (and therefore force) can be increased may however be limited by motor control strategies. Specifically, a suboptimal inter-muscular coordination may introduce noise (or errors) in the timing of muscle activation, and a slower increase in muscle activation (and thus force) may reduce the sensitivity of jump performance to these errors [37,42]. As a result, individuals with a high RFD_ISO_ but poor inter-muscular coordination may not be able to use their ability to rapidly increase muscle activation during the jump, resulting in low correlations. The associations between single-joint RFD_ISO_ values and CMJ outcomes were in line with, but generally slightly lower for unilateral CMJ's as compared to bilateral CMJ's (Supplementary file 2). This has previously also been observed [22], and could reflect a greater contribution of other factors such as technique and balance, as well as an influence of bilateral deficit [43], which adds further indirect evidence to the effects of inter-muscular coordination ability on the transfer of RFD_ISO_ to jumping RFD. Due to the high similarity between bilateral and unilateral findings, the remaining discussion will primarily focus on the bilateral findings.

Single-joint RFD_ISO_ showed trivial associations with SJ and CMJ duration or the duration of specific phases (Table SI & SII). Similarly, multiple single-joint RFD_ISO_ metrics in a regression model could explain only ∼2–3% of the variance in SJ and CMJ duration, respectively. Since participants were instructed to maximize both jump height and minimize jump duration, a higher single-joint RTD might therefore have a larger effect on improving jump height (∼20–26% explained variance) as compared to shortening jump duration in a setting where both aspects are of importance. Finally, single-joint RFD_ISO_ also showed small to moderate associations with CMJ and SJ jump height. These findings are in agreement with authors reporting mostly trivial to small associations between single-joint RTD development and jump height [[10], [11], [12],^15^,^16^], or report conflicting findings depending on the parameter investigated [12,[20], [21], [22]]. Computational models have estimated that the work done by the hip extensors contributes most to vertical CMJ performance (∼38%), followed by the knee (∼32%) and ankle (∼30%) extensors [44]. Similarly, in a comparison between better and poorer jumpers, Vanezis and Lees [45] found that better CM jumpers generated particularly more work at the hip (0.72 ​J/kg), followed by the ankle (0.39 ​J/kg) and knee (0.26 ​J/kg). Our linear regression model however showed that ankle extensors RTD explained most of the variance in CMJ height, followed by knee extensors RTD and only finally hip extensors RTD, with the latter having a non-significant contribution to the model. Similar findings were observed for the SJ. This discrepancy between the importance of work done by the hip extensors and a relatively low explained variance by hip extensor RTD in the current study could be because RTD is only one factor that explains the amount of work done, with other factors such as peak torque and intermuscular coordination also being important factors [42]. Additionally, the CM may have provided individuals with a relatively long time to build up an active state of the hip extensors [37], which could reduce the important of having a high RFD of the hip extensors in the CMJ. Yet this latter reason does not explain why hip extensors RTD did not contribute significantly to jump height in the SJ where there is no CM that might be used to compensate for a relatively slower hip extensor RTD. Another explanation for this finding could therefore be related to the relatively non-specific joint configuration and contraction mode in which the RTD assessments were performed (Fig. 1). Specifically, isometric muscle actions may differ in the neural activation from concentric contractions [46] and hereby affect RFD. Further, isometric assessments may restrict muscle bulging and hereby limit architectural gearing from contributing to RFD, which can further reduce the transfer between isometric and concentric RFD [47]. In further support of the effect of joint configuration, a previous study reported no significant differences between RFD in the SJ and CMJ and isometric leg press RFD, which the authors attributed to the largely similar joint positions of the assessments [40].

There are several limitations to this study. First, we only used single-joint isometric strength measurements, of which some were performed in non-specific joint configurations, which decreases the specificity and therefore correlations with regard to jumping. While multi-joint assessments are likely to exhibit more similarity to the multi-joint nature of jumping, multi-joint assessments do not provide information about the relative importance of each involved joint/muscle group and we therefore decided to rather combine single-joint assessments in a linear regression approach. Related to this, the reliability of our RFD measures was poor to moderate, and this could also have impacted our ability to establish correlations. Nevertheless, the used positions and equipment facilitated large-scale data collection and may better reflect how practitioners collected data in practice among large groups of individuals than other procedures often used in research settings. Further, we used the average of the two best repetitions in correlations and regression analysis to reduce the influence of the lower reliability. Second, RTD assessments involved fast, but sustained contractions, while it has been suggested that fast contraction pulses offer a more valid approach to RFD assessment [28]. Third, we combined individuals with different training backgrounds and different sex in all analyses. However, RFD and jump outcomes are known to differ between individuals with different training backgrounds and sex [23]. Indeed, when data were analyzed separately for males and females, males generally showed higher jump heights and a higher rate of force development during jumping and the isometric assessments. However, the correlations between all outcomes were generally similar for the combined group, and males and females separately (see supplementary file 2), suggesting this did not substantially impact our findings. Similarly, differences in the age and thus maturation of participants (e.g. 15.9 years for tennis players vs 19.8 years for physical education students) could also have influenced our findings due to potential differences in for example muscle coordination and structural musculotendinous properties between participants. Fourth, the mechanistic linkage of braking RFD during the CMJ with the net propulsive impulse is not clear and may also partially explain the low correlations between RFD and jump outcomes during the CMJ. Nevertheless, brake phase RFD during the CMJ is often assessed in other studies and practice, thus necessitating the need for investigating this outcome. Moreover, similar associations were observed during the SJ where there is a direct link between the RFD and net propulsive impulse, thus also adding some confidence to the findings observed for the CMJ. Finally, the sampling rate differed between the sensors used to assess knee and ankle (1000 ​Hz) and hip (450 ​Hz) RFD. Although this could therefore impact the accuracy of the hip RFD values, we expect the effect on the obtained correlation to be small since the same sample rate was used across all individuals.

The finding of small positive correlations between jumping RFD during jumping and jump height, as well as a moderate correlation between jumping RFD and SJ duration implies that improving of RFD could benefit jump performance. However, the mostly small correlation between single-joint isometric and dynamic multi-joint (i.e., jumping) RFD suggests that assessment of single-joint isometric RFD provides only limited information regarding the RFD in more complex sports-related movements. Additionally, this finding also questions the relevance of improving single-joint isometric RFD for improving dynamic multi-joint RFD [1].

## 5 Conclusion

In conclusion, single-joint rate of force development shows mostly small correlations with jumping rate of force development and jump height, and trivial associations with jump duration. A combination of single-joint rate of force development from different muscle groups however explained a small to moderate variance in jump height (∼23–28%), duration (∼2–3%), and RFD during jumping (∼19–28%). Finally, rate of force development during jumping showed a small positive association with jump height and trivial to moderate negative correlations with jump duration.

## Author contributions

Devising research (BVH, ZK, NS), data collection (ZK, DS), data analysis (BVH, ZK), manuscript writing (BHV), editing the manuscript (all authors). All authors provided suggestions, revisions and edits to the manuscript and approved the final version.

## Funding info

No funding received.

## Ethical statement

The participants were informed about the testing procedures and signed an informed consent before participation. The experiment was approved by the Republic of Slovenia National Medical Ethics Committee (approval no. 0120–99/2018/5).

## Declaration of competing interest

The authors declare that they have no known competing financial interests or personal relationships that could have appeared to influence the work reported in this paper.

## References

[1] Van Hooren B., Bosch F., Meijer K. (2017). Can resistance training enhance the rapid force development in unloaded dynamic isoinertial multi-joint movements? A systematic review. J Strength Cond Res.

[2] Kirby T.J., McBride J.M., Haines T.L., Dayne A.M. (2011). Relative net vertical impulse determines jumping performance. J Appl Biomech.

[3] McLellan C.P., Lovell D.I., Gass G.C. (2011). The role of rate of force development on vertical jump performance. J Strength Cond Res.

[4] Marques M.C., Van den Tillaar R., Izquierdo M., Moir G.L., Sanchez-Medina L., Gonzalez-Badillo J.J. (2014). The reliability of force-time variables recorded during vertical jump performance and their relationship with jump height in power trained athletes. International SportMed Journal.

[5] Souza A.A., Bottaro M., Rocha V.A. (2020). Reliability and test-retest agreement of mechanical variables obtained during countermovement jump. International journal of exercise science.

[6] Wilson G.J., Lyttle A.D., Ostrowski K.J., Murphy A.J. (1995). Assessing dynamic performance: A comparison of rate of force development tests. J Strength Cond Res.

[7] Kawamori N., Rossi S.J., Justice B.D., Haff E.E., Pistilli E.E., O’Bryant H.S. (2006). Peak force and rate of force development during isometric and dynamic mid-thigh clean pulls performed at various intensities. J Strength Cond Res.

[8] Haff G.G., Stone M., O’Bryant H.S., Harman E., Dinan C., Johnson R. (1997). Force-time dependent characteristics of dynamic and isometric muscle actions. J Strength Cond Res.

[9] Suchomel T.J., Sole C.J., Bellon C.R., Stone M.H. (2020). Dynamic Strength Index: Relationships with Common Performance Variables and Contextualization of Training Recommendations. J Hum Kinet.

[10] Thompson B.J., Ryan E.D., Sobolewski E.J., Smith D.B., Akehi K., Conchola E.C. (2013). Relationships between rapid isometric torque characteristics and vertical jump performance in division i collegiate american football players: Influence of body mass normalization. J Strength Cond Res.

[11] Chang E., Norcross M.F., Johnson S.T., Kitagawa T., Hoffman M. (2015). Relationships between explosive and maximal triple extensor muscle performance and vertical jump height. J Strength Cond Res.

[12] McKinlay B.J., Wallace P.J., Dotan R., Long D., Tokuno C., Gabriel D.A. (2017). Isometric and dynamic strength and neuromuscular attributes as predictors of vertical jump performance in 11- to 13-year-old male athletes. Appl Physiol Nutr Metab.

[13] West D.J., Owen N.J., Jones M.R., Bracken R.M., Cook C.J., Cunningham D.J. (2011). Relationships between force–time characteristics of the isometric midthigh pull and dynamic performance in professional rugby league players. J Strength Cond Res.

[14] Kraska J.M., Ramsey M.W., Haff G.G., Fethke N., Sands W.A., Stone M.E. (2009). Relationship between strength characteristics and unweighted and weighted vertical jump height. Int J Sports Physiol Perform.

[15] McErlain-Naylor S., King M., Pain M.T. (2014). Determinants of countermovement jump performance: a kinetic and kinematic analysis. J Sports Sci.

[16] Gillen Z.M., Shoemaker M.E., McKay B.D., Bohannon N.A., Gibson S.M., Cramer J.T. (2020). Leg extension strength, explosive strength, muscle activation, and growth as predictors of vertical jump performance in youth athletes. Journal of Science in Sport and Exercise.

[17] Thomas C., Jones P.A., Rothwell J., Chiang C.Y., Comfort P. (2015). An Investigation Into the Relationship Between Maximum Isometric Strength and Vertical Jump Performance. J Strength Cond Res.

[18] Nuzzo J.L., McBride J.M., Cormie P., McCaulley G.O. (2008). Relationship between countermovement jump performance and multijoint isometric and dynamic tests of strength. J Strength Cond Res.

[19] McGuigan M.R., Winchester J.B. (2008). The relationship between isometric and dynamic strength in college football players. J Sports Sci Med.

[20] Laett C.T., Cossich V., Goes R.A., Gavilão U., Rites A., de Oliveira C.G. (2021). Relationship between vastus lateralis muscle ultrasound echography, knee extensors rate of torque development, and jump height in professional soccer athletes. Sport Sciences for Health.

[21] Khamoui A.V., Brown L.E., Nguyen D., Uribe B.P., Coburn J.W., Noffal G.J. (2011). Relationship between force-time and velocity-time characteristics of dynamic and isometric muscle actions. The Journal of Strength and Conditioning Research.

[22] Kozinc Z., Šarabon N. (2021). Measurements of Lower-limb Isometric Single-joint Maximal Voluntary Torque and Rate of Torque Development Capacity Offer Limited Insight into Vertical Jumping Performance. Measurement in Physical Education and Exercise Science.

[23] Maffiuletti N.A., Aagaard P., Blazevich A.J., Folland J., Tillin N., Duchateau J. (2016). Rate of force development: physiological and methodological considerations. Eur J Appl Physiol.

[24] Haff G.G., Ruben R.P., Lider J., Twine C., Cormie P. (2015). A comparison of methods for determining the rate of force development during isometric midthigh clean pulls. J Strength Cond Res.

[25] Drake D., Kennedy R.A., Wallace E.S. (2019). Multi-joint rate of force development testing protocol affects reliability and the smallest detectible difference. J Sports Sci.

[26] Guppy S.N., Kotani Y., Brady C.J., Connolly S., Comfort P., Haff G.G. (2022). The Reliability and Magnitude of Time-Dependent Force-Time Characteristics During the Isometric Midthigh Pull Are Affected by Both Testing Protocol and Analysis Choices. J Strength Cond Res.

[27] McCormick B., MacMahon C., Talpey S., James L. (2022). The Influence of Instruction on Isometric Mid-Thigh Pull Force-Time Variables. International Journal of Strength and Conditioning.

[28] Sahaly R., Vandewalle H., Driss T., Monod H. (2001). Maximal voluntary force and rate of force development in humans--importance of instruction. Eur J Appl Physiol.

[29] Caruso J., McEnroe C., Vanhoove A., Chen L., Vargas L., Carter K. (2019). Performance-based correlates to vertical jump height and power values in women. Isokinet Exerc Sci.

[30] VanVoorhis C.W., Morgan B.L. (2007). Understanding power and rules of thumb for determining sample sizes. Tutorials in quantitative methods for psychology.

[31] Laffaye G., Wagner P.P., Tomblesion T.I.L. (2014). Countermovement jump height: Gender and sport-specific differences in the forcetime variables. J Strength Cond Res.

[32] Ebben W.P., Flanagan E.P., Jensen R.J. (2007). Gender similarities in rate of force development and time to takeoff during the countermovement jump. Journal of Exercise Physiolology.

[33] Sarabon N., Knezevic M.O., Mirkov M.D., Smajla D. (2020). Introduction of dynamic rate-of-force development scaling factor in progressive drop jumps. J Biomech.

[34] Pupo J.D., Detanico D., dos Santos S.G. (2012(May). Kinetic parameters as determinants of vertical jump performance. Brazilian Journal of Kinanthropomerty and Human Performance.

[35] Marcora S., Miller M.K. (2000). The effect of knee angle on the external validity of isometric measures of lower body neuromuscular function. J Sports Sci.

[36] Barker L.A., Harry J.R., Mercer J.A. (2018). Relationships Between Countermovement Jump Ground Reaction Forces and Jump Height, Reactive Strength Index, and Jump Time. J Strength Cond Res.

[37] Van Hooren B., Zolotarjova J. (2017). The Difference Between Countermovement and Squat Jump Performances: A Review of Underlying Mechanisms With Practical Applications. J Strength Cond Res.

[38] McMahon J.J., Suchomel T.J., Lake J.P., Comfort P. (2018). Understanding the Key Phases of the Countermovement Jump Force-Time Curve. Strength Cond J.

[39] McHugh M.P., Hickok M., Cohen J.A., Virgile A., Connolly D.A. (2021). Is there a biomechanically efficient vertical ground reaction force profile for countermovement jumps?. Translational Sports Medicine.

[40] Martinopoulou K., Donti O., Sands W.A., Terzis G., Bogdanis G.C. (2022). Evaluation of The Isometric and Dynamic Rates of Force Development in Multi-Joint Muscle Actions. J Hum Kinet.

[41] Hernández G., Domínguez D., Moreno J., Til L., Capdevila L., Pedret C. (2016). Patellar tendon analysis by ultrasound tissue characterization; comparison between professional and amateur basketball players. Asymptomatic versus symptomatic. Apunts Med Esport.

[42] Bobbert M.F., van Zandwijk J.P. (1999). Sensitivity of vertical jumping performance to changes in muscle stimulation onset times: a simulation study. Biol Cybern.

[43] Bobbert M.F., de Graaf W.W., Jonk J.N., Casius L.J. (1985). Explanation of the bilateral deficit in human vertical squat jumping. J Appl Physiol.

[44] Bobbert M.F., Mackay M., Schinkelshoek D., Huijing P.A., van Ingen Schenau G.J. (1986). Biomechanical analysis of drop and countermovement jumps. Eur J Appl Physiol Occup Physiol.

[45] Vanezis A., Lees A. (2007). A biomechanical analysis of good and poor performers of the vertical jump. Ergonomics.

[46] Murphy A.J., Wilson G.J. (1996). Poor correlations between isometric tests and dynamic performance: relationship to muscle activation. Eur J Appl Physiol Occup Physiol.

[47] Van Hooren B., Aagaard P., Monte A., Blazevich A. (2022). Role of pennation angle in rapid force development during isometric and dynamic muscle actions. Exerc Sport Sci Rev.

