# Supplementary material for: Isometric single-joint rate of force development shows trivial to small associations with jumping rate of force development, jump height, and propulsive duration
Source: JSAMS Plus. 2022 Oct 22;1:100006. doi: 10.1016/j.jsampl.2022.100006 (PMC13008453; doi:10.1016/j.jsampl.2022.100006)
Supplement: Multimedia component 2 [file mmc2.docx]

**Table SI. Mean ± SD for all assessed variables for males only**

| **Outcome** | | **Mean ± SD bilateral outcomes** | **Mean ± SD left leg outcomes** | **Mean ± SD right leg outcomes** |
| --- | --- | --- | --- | --- |
| **Squat jump** |  | |  |  |
| Jump height [m] | | 0.28 ± 0.05 | n.a. | n.a. |
| Propulsion time [s] | | 0.34 ± 0.05 | n.a. | n.a. |
| Time to maximum force [s] | | 0.24 ± 0.05 | n.a. | n.a. |
| Peak RFD [N/s] | | 9761 ± 5264 | n.a. | n.a. |
| **Countermovement jump** |  | |  |  |
| Jump height [m] | | 0.31 ± 0.05 | 0.14 ± 0.04 | 0.15 ± 0.04 |
| Jump time [s] | | 0.71 ± 0.07 | 0.76 ± 0.09 | 0.77 ± 0.09 |
| Countermovement time [s] | | 0.46 ± 0.05 | 0.46 ± 0.06 | 0.47 ± 0.05 |
| Propulsion time [s] | | 0.26 ± 0.03 | 0.30 ± 0.04 | 0.31 ± 0.04 |
| Braking time [s] | | 0.27 ± 0.04 | 0.29 ± 0.05 | 0.29 ± 0.05 |
| Time to maximum force [s] | | 0.50 ± 0.08 | 0.56 ± 0.12 | 0.58 ± 0.11 |
| Peak RFD [N/s] | | 15052 ± 5217 | 8112 ± 4991 | 9920 ± 4431 |
| **Isometric Strength** |  | |  |  |
| Knee extensors peak RTD [Nm/s] | | n.a. | 1051 ± 439 | 1021 ± 227 |
| Ankle plantar flexors peak RTD [Nm/s] | | 1639 ± 511 | n.a. | n.a. |
| Hip extensors peak RTD [Nm/s] | | n.a. | 1140 ± 553 | 1206 ± 562 |

RFD = rate of force development; RTD = rate of torque development

**Table SII. Mean ± SD for all assessed variables for females only**

| **Outcome** | | **Mean ± SD bilateral outcomes** | **Mean ± SD left leg outcomes** | **Mean ± SD right leg outcomes** |
| --- | --- | --- | --- | --- |
| **Squat jump** |  | |  |  |
| Jump height [m] | | 0.22 ± 0.04 | n.a. | n.a. |
| Propulsion time [s] | | 0.36 ± 0.06 | n.a. | n.a. |
| Time to maximum force [s] | | 0.26 ± 0.06 | n.a. | n.a. |
| Peak RFD [N/s] | | 6331 ± 2686 | n.a. | n.a. |
| **Countermovement jump** |  | |  |  |
| Jump height [m] | | 0.23 ± 0.03 | 0.10 ± 0.03 | 0.11 ± 0.03 |
| Jump time [s] | | 0.72 ± 0.09 | 0.77 ± 0.10 | 0.77 ± 0.10 |
| Countermovement time [s] | | 0.45 ± 0.05 | 0.46 ± 0.06 | 0.46 ± 0.06 |
| Propulsion time [s] | | 0.27 ± 0.03 | 0.31 ± 0.04 | 0.31 ± 0.05 |
| Braking time [s] | | 0.28 ± 0.05 | 0.28 ± 0.05 | 0.28 ± 0.05 |
| Time to maximum force [s] | | 0.48 ± 0.10 | 0.53 ± 0.13 | 0.55 ± 0.14 |
| Peak RFD [N/s] | | 10874 ± 3572 | 6601 ± 4347 | 7498 ± 3162 |
| **Isometric Strength** |  | |  |  |
| Knee extensors peak RTD [Nm/s] | | n.a. | 765 ± 282 | 741 ± 301 |
| Ankle plantar flexors peak RTD [Nm/s] | | 1119 ± 385 | n.a. | n.a. |
| Hip extensors peak RTD [Nm/s] | | n.a. | 785 ± 379 | 840 ± 379 |

RFD = rate of force development; RTD = rate of torque development

**Table SIII. Correlations between rate of force development and bilateral SJ metrics for males and females combined**

| **Variable** | **SJ height [m]** | | | **SJ propulsion time [s]** | | **SJ time to max force [s]** | | **SJ peak RFD [N/s]** | | |
| --- | --- | --- | --- | --- | --- | --- | --- | --- | --- | --- |
| SJ height [m] | — |  |  | |  |  |  |  |  |  |
|  | — |  |  | |  |  |  |  |  |  |
| SJ propulsion time [s] | -0.226 | *** | — | |  |  |  |  |  |  |
|  | < .001 |  | — | |  |  |  |  |  |  |
| SJ time to max force [s] | -0.111 | * | 0.945 | | *** | — |  |  |  |  |
|  | 0.046 |  | < .001 | |  | — |  |  |  |  |
| SJ peak RFD [N/s] | 0.206 | *** | -0.639 | | *** | -0.637 | *** | — |  |  |
|  | < .001 |  | < .001 | |  | < .001 |  | — |  |  |
| Left knee extensors peak RTD [Nm/s] | 0.225 | *** | -0.042 | |  | -0.038 |  | 0.148 | ** |  |
|  | < .001 |  | 0.455 | |  | 0.501 |  | 0.009 |  |  |
| Right knee extensors peak RTD [Nm/s] | 0.197 | *** | -0.022 | |  | -0.014 |  | 0.128 | * |  |
|  | < .001 |  | 0.698 | |  | 0.802 |  | 0.023 |  |  |
| Ankle plantar flexors peak RTD [Nm/s] | 0.274 | *** | -0.092 | |  | -0.074 |  | 0.334 | *** |  |
|  | < .001 |  | 0.098 | |  | 0.187 |  | < .001 |  |  |
| Left hip extensors peak RTD [Nm/s] | 0.183 | ** | -0.113 | | * | -0.097 |  | 0.221 | *** |  |
|  | 0.001 |  | 0.043 | |  | 0.083 |  | < .001 |  |  |
| Right hip extensors peak RTD [Nm/s] | 0.150 | ** | -0.075 | |  | -0.076 |  | 0.223 | *** |  |
|  | 0.007 |  | 0.183 | |  | 0.178 |  | < .001 |  |  |

* p < .05, ** p < .01, *** p < .001

**Table SIV. Correlations between rate of force development and bilateral SJ metrics for males only**

| **Variable** | **SJ height [m]** | | **SJ propulsion time [s]** | | **SJ time to max force [s]** | | **SJ peak RFD [N/s]** | |  |
| --- | --- | --- | --- | --- | --- | --- | --- | --- | --- |
| SJ height [m] | | — | |  | |  | |  | |
|  |  | — | |  | |  | |  | |
| SJ propulsion time [s] | | -.233^**^ | | — | |  | |  | |
|  |  | .001 | | — | |  | |  | |
| SJ time to max force [s] | | -.099 | | .952^**^ | | — | |  | |
|  |  | .168 | | .000 | | — | |  | |
| SJ peak RFD [N/s] | | .206^**^ | | -.675^**^ | | -.684^**^ | | — | |
|  |  | .004 | | .000 | | .000 | | — | |
| Left knee extensors peak RTD [Nm/s] | | .202^**^ | | -.062 | | -.039 | | .170^*^ | |
|  |  | .005 | | .394 | | .593 | | .019 | |
| Right knee extensors peak RTD [Nm/s] | | .183^*^ | | -.018 | | .003 | | .150^*^ | |
|  |  | .011 | | .801 | | .968 | | .039 | |
| Ankle plantar flexors peak RTD [Nm/s] | | .302^**^ | | -.171^*^ | | -.156^*^ | | .387^**^ | |
|  |  | .000 | | .016 | | .029 | | .000 | |
| Left hip extensors peak RTD [Nm/s] | | .197^**^ | | -.157^*^ | | -.154^*^ | | .250^**^ | |
|  |  | .006 | | .029 | | .032 | | .000 | |
| Right hip extensors peak RTD [Nm/s] | | .195^**^ | | -.087 | | -.094 | | .213^**^ | |
|  |  | .007 | | .232 | | .193 | | .003 | |

**Table SV. Correlations between rate of force development and bilateral SJ metrics for females only**

| **Variable** | **SJ height [m]** | | **SJ propulsion time [s]** | | **SJ time to max force [s]** | | **SJ peak RFD [N/s]** | |  |
| --- | --- | --- | --- | --- | --- | --- | --- | --- | --- |
| SJ height [m] | | — | |  | |  | |  | |
|  |  | — | |  | |  | |  | |
| SJ propulsion time [s] | | -.231^**^ | | — | |  | |  | |
|  |  | .009 | | — | |  | |  | |
| SJ time to max force [s] | | -.146 | | .936^**^ | | — | |  | |
|  |  | .100 | | .000 | | — | |  | |
| SJ peak RFD [N/s] | | .217^*^ | | -.699^**^ | | -.681^**^ | | — | |
|  |  | .014 | | .000 | | .000 | | — | |
| Left knee extensors peak RTD [Nm/s] | | .303^**^ | | -.005 | | -.041 | | .045 | |
|  |  | .001 | | .954 | | .651 | | .617 | |
| Right knee extensors peak RTD [Nm/s] | | .238^**^ | | -.030 | | -.047 | | .042 | |
|  |  | .008 | | .737 | | .605 | | .641 | |
| Ankle plantar flexors peak RTD [Nm/s] | | .191^*^ | | .044 | | .065 | | .143 | |
|  |  | .032 | | .623 | | .465 | | .110 | |
| Left hip extensors peak RTD [Nm/s] | | .139 | | -.039 | | .001 | | .102 | |
|  |  | .118 | | .661 | | .992 | | .251 | |
| Right hip extensors peak RTD [Nm/s] | | .006 | | -.059 | | -.048 | | .274^**^ | |
|  |  | .947 | | .513 | | .590 | | .002 | |

**Table SVI. Correlations between rate of force development and bilateral CMJ metrics for males and females combined**

| **Variable** | | **CMJ height [m]** | | **CMJ jump time [s]** | | **CMJ CM time [s]** | | **CMJ propulsion time [s]** | | **CMJ time to max force [s]** | | **CMJ peak RFD [N/s]** | |
| --- | --- | --- | --- | --- | --- | --- | --- | --- | --- | --- | --- | --- | --- |
| CMJ height [m] |  | — |  |  |  |  |  |  |  |  |  |  |  |
|  |  | — |  |  |  |  |  |  |  |  |  |  |  |
| CMJ jump time [s] |  | 0.006 |  | — |  |  |  |  |  |  |  |  |  |
|  |  | 0.917 |  | — |  |  |  |  |  |  |  |  |  |
| CMJ CM time [s] |  | 0.113 | * | 0.946 | *** | — |  |  |  |  |  |  |  |
|  |  | 0.043 |  | < .001 |  | — |  |  |  |  |  |  |  |
| CMJ propulsion time [s] |  | -0.166 | ** | 0.863 | *** | 0.639 | *** | — |  |  |  |  |  |
|  |  | 0.003 |  | < .001 |  | < .001 |  | — |  |  |  |  |  |
| CMJ time to max force [s] |  | 0.049 |  | 0.681 | *** | 0.686 | *** | 0.475 | *** | — |  |  |  |
|  |  | 0.382 |  | < .001 |  | < .001 |  | < .001 |  | — |  |  |  |
| CMJ peak RFD [N/s] |  | 0.400 | *** | -0.339 | *** | -0.277 | *** | -0.354 | *** | -0.358 | *** | — |  |
|  |  | < .001 |  | < .001 |  | < .001 |  | < .001 |  | < .001 |  | — |  |
| Left knee extensors peak RTD [Nm/s] |  | 0.412 | *** | -0.081 |  | -0.046 |  | -0.120 | * | -0.089 |  | 0.420 | *** |
|  |  | < .001 |  | 0.151 |  | 0.419 |  | 0.035 |  | 0.115 |  | < .001 |  |
| Right knee extensors peak RTD [Nm/s] |  | 0.398 | *** | -0.085 |  | -0.043 |  | -0.145 | * | -0.092 |  | 0.438 | *** |
|  |  | < .001 |  | 0.133 |  | 0.442 |  | 0.011 |  | 0.103 |  | < .001 |  |
| Ankle plantar flexors peak RTD [Nm/s] |  | 0.509 | *** | -0.054 |  | 0.027 |  | -0.158 | ** | -4.473e-4 |  | 0.497 | *** |
|  |  | < .001 |  | 0.330 |  | 0.629 |  | 0.004 |  | 0.994 |  | < .001 |  |
| Left hip extensors peak RTD [Nm/s] |  | 0.388 | *** | 4.264e-5 |  | 0.044 |  | -0.076 |  | -0.023 |  | 0.396 | *** |
|  |  | < .001 |  | 0.999 |  | 0.431 |  | 0.174 |  | 0.677 |  | < .001 |  |
| Right hip extensors peak RTD [Nm/s] |  | 0.408 | *** | 0.114 | * | 0.155 | ** | 0.031 |  | 0.063 |  | 0.386 | *** |
|  |  | < .001 |  | 0.041 |  | 0.006 |  | 0.582 |  | 0.263 |  | < .001 |  |

* p < .05, ** p < .01, *** p < .001

**Table SVII. Correlations between rate of force development and bilateral CMJ metrics for males only**

| **Variable** | **CMJ height [m]** | **CMJ jump time [s]** | **CMJ CM time [s]** | **CMJ propulsion time [s]** | **CMJ time to max force [s]** | **CMJ peak RFD [N/s]** |
| --- | --- | --- | --- | --- | --- | --- |
| CMJ height [m] | — |  |  |  |  |  |
|  | — |  |  |  |  |  |
| CMJ jump time [s] | .052 | — |  |  |  |  |
|  | .467 | — |  |  |  |  |
| CMJ CM time [s] | .126 | .944^**^ | — |  |  |  |
|  | .078 | .000 | — |  |  |  |
| CMJ propulsion time [s] | -.099 | .869^**^ | .656^**^ | — |  |  |
|  | .168 | .000 | .000 | — |  |  |
| CMJ time to max force [s] | .011 | .632^**^ | .632^**^ | .484^**^ | — |  |
|  | .878 | .000 | .000 | .000 | — |  |
| CMJ peak RFD [N/s] | .245^**^ | -.321^**^ | -.279^**^ | -.314^**^ | -.373^**^ | — |
|  | .001 | .000 | .000 | .000 | .000 | — |
| Left knee extensors peak RTD [Nm/s] | .248^**^ | -.040 | -.020 | -.072 | -.075 | .319^**^ |
|  | .001 | .584 | .787 | .328 | .303 | .000 |
| Right knee extensors peak RTD [Nm/s] | .242^**^ | -.068 | -.043 | -.103 | -.131 | .348^**^ |
|  | .001 | .351 | .555 | .159 | .070 | .000 |
| Ankle plantar flexors peak RTD [Nm/s] | .326^**^ | -.024 | .034 | -.102 | -.006 | .409^**^ |
|  | .000 | .741 | .631 | .153 | .939 | .000 |
| Left hip extensors peak RTD [Nm/s] | .255^**^ | .009 | .046 | -.061 | -.052 | .343^**^ |
|  | .000 | .904 | .526 | .402 | .472 | .000 |
| Right hip extensors peak RTD [Nm/s] | .311^**^ | .154^*^ | .185^**^ | .071 | .061 | .319^**^ |
|  | .000 | .032 | .010 | .325 | .397 | .000 |

**Table SVIII. Correlations between rate of force development and bilateral CMJ metrics for females only**

| **Variable** | | **CMJ height [m]** | | **CMJ jump time [s]** | | **CMJ CM time [s]** | | **CMJ propulsion time [s]** | | **CMJ time to max force [s]** | | **CMJ peak RFD [N/s]** | |
| --- | --- | --- | --- | --- | --- | --- | --- | --- | --- | --- | --- | --- | --- |
| CMJ height [m] | — | |  | |  | |  | |  | |  | |  |
|  | — | |  | |  | |  | |  | |  | |  |
| CMJ jump time [s] | -.005 | | — | |  | |  | |  | |  | |  |
|  | .954 | | — | |  | |  | |  | |  | |  |
| CMJ CM time [s] | .067 | | .954^**^ | | — | |  | |  | |  | |  |
|  | .458 | | .000 | | — | |  | |  | |  | |  |
| CMJ propulsion time [s] | -.111 | | .867^**^ | | .650^**^ | | — | |  | |  | |  |
|  | .216 | | .000 | | .000 | | — | |  | |  | |  |
| CMJ time to max force [s] | -.002 | | .739^**^ | | .743^**^ | | .508^**^ | | — | |  | |  |
|  | .979 | | .000 | | .000 | | .000 | | — | |  | |  |
| CMJ peak RFD [N/s] | .116 | | -.455^**^ | | -.444^**^ | | -.370^**^ | | -.570^**^ | | — | |  |
|  | .199 | | .000 | | .000 | | .000 | | .000 | | — | |  |
| Left knee extensors peak RTD [Nm/s] | .335^**^ | | -.192^*^ | | -.205^*^ | | -.080 | | -.264^**^ | | .342^**^ | |  |
|  | .000 | | .032 | | .022 | | .381 | | .003 | | .000 | |  |
| Right knee extensors peak RTD [Nm/s] | .307^**^ | | -.139 | | -.137 | | -.099 | | -.157 | | .340^**^ | |  |
|  | .001 | | .125 | | .129 | | .279 | | .082 | | .000 | |  |
| Ankle plantar flexors peak RTD [Nm/s] | .256^**^ | | -.126 | | -.083 | | -.097 | | -.136 | | .281^**^ | |  |
|  | .004 | | .157 | | .355 | | .284 | | .127 | | .001 | |  |
| Left hip extensors peak RTD [Nm/s] | .200^*^ | | -.015 | | -.027 | | .043 | | -.080 | | .158 | |  |
|  | .025 | | .867 | | .758 | | .636 | | .367 | | .077 | |  |
| Right hip extensors peak RTD [Nm/s] | .115 | | .076 | | .059 | | .136 | | -.015 | | .179^*^ | |  |
|  | .204 | | .395 | | .507 | | .132 | | .869 | | .046 | |  |

**Table SIX. Correlations between rate of force development and unilateral (left leg) CMJ metrics for males and females combined**

| **Variable** | | **CMJ left jump height [m]** | | **CMJ left jump time [s]** | | **CMJ left CM time [s]** | | **CMJ left propulsion time [s]** | | **CMJ left time to max force [s]** | | **CMJ left max RFD [N/s]** | |
| --- | --- | --- | --- | --- | --- | --- | --- | --- | --- | --- | --- | --- | --- |
| CMJ left jump height [m] |  | — |  |  |  |  |  |  |  |  |  |  |  |
|  |  | — |  |  |  |  |  |  |  |  |  |  |  |
| CMJ left jump time [s] |  | -0.093 |  | — |  |  |  |  |  |  |  |  |  |
|  |  | 0.096 |  | — |  |  |  |  |  |  |  |  |  |
| CMJ left CM time [s] |  | -0.056 |  | 0.945 | *** | — |  |  |  |  |  |  |  |
|  |  | 0.316 |  | < .001 |  | — |  |  |  |  |  |  |  |
| CMJ left propulsion time [s] |  | -0.127 | * | 0.879 | *** | 0.674 | *** | — |  |  |  |  |  |
|  |  | 0.022 |  | < .001 |  | < .001 |  | — |  |  |  |  |  |
| CMJ left time to max force [s] |  | 0.076 |  | 0.735 | *** | 0.755 | *** | 0.557 | *** | — |  |  |  |
|  |  | 0.171 |  | < .001 |  | < .001 |  | < .001 |  | — |  |  |  |
| CMJ left max RFD [N/s] |  | 0.095 |  | -0.466 | *** | -0.470 | *** | -0.366 | *** | -0.638 | *** | — |  |
|  |  | 0.089 |  | < .001 |  | < .001 |  | < .001 |  | < .001 |  | — |  |
| Knee extensors left max RTD [Nm/s] |  | 0.348 | *** | -0.032 |  | -0.056 |  | 0.009 |  | -0.065 |  | 0.272 | *** |
|  |  | < .001 |  | 0.572 |  | 0.327 |  | 0.869 |  | 0.253 |  | < .001 |  |
| Ankle plantar flexors max RTD [Nm/s] |  | 0.463 | *** | 0.001 |  | 0.022 |  | -0.029 |  | 0.024 |  | 0.258 | *** |
|  |  | < .001 |  | 0.985 |  | 0.698 |  | 0.601 |  | 0.661 |  | < .001 |  |
| Hip extensors left max RTD [Nm/s] |  | 0.276 | *** | 0.063 |  | 0.057 |  | 0.060 |  | 0.038 |  | 0.227 | *** |
|  |  | < .001 |  | 0.257 |  | 0.305 |  | 0.287 |  | 0.497 |  | < .001 |  |

* p < .05, ** p < .01, *** p < .001

**Table SIX. Correlations between rate of force development and unilateral (right leg) CMJ metrics for males and females combined**

| **Variable** | | **CMJ right** **jump height [m]** | | **CMJ right** **jump time [s]** | | **CMJ right** **CM time [s]** | | **CMJ right** **propulsion time [s]** | | **CMJ right** **time to max force [s]** | | **CMJ right** **max RFD [N/s]** | |
| --- | --- | --- | --- | --- | --- | --- | --- | --- | --- | --- | --- | --- | --- |
| CMJ right jump height [m] |  | — |  |  |  |  |  |  |  |  |  |  |  |
|  |  | — |  |  |  |  |  |  |  |  |  |  |  |
| CMJ right jump time [s] |  | -0.103 |  | — |  |  |  |  |  |  |  |  |  |
|  |  | 0.065 |  | — |  |  |  |  |  |  |  |  |  |
| CMJ right CM time [s] |  | -0.040 |  | 0.933 | *** | — |  |  |  |  |  |  |  |
|  |  | 0.471 |  | < .001 |  | — |  |  |  |  |  |  |  |
| CMJ right propulsion time [s] |  | -0.163 | ** | 0.886 | *** | 0.659 | *** | — |  |  |  |  |  |
|  |  | 0.003 |  | < .001 |  | < .001 |  | — |  |  |  |  |  |
| CMJ right time to max force [s] |  | 0.102 |  | 0.750 | *** | 0.785 | *** | 0.555 | *** | — |  |  |  |
|  |  | 0.065 |  | < .001 |  | < .001 |  | < .001 |  | — |  |  |  |
| CMJ right max RFD [N/s] |  | 0.206 | *** | -0.369 | *** | -0.416 | *** | -0.234 | *** | -0.433 | *** | — |  |
|  |  | < .001 |  | < .001 |  | < .001 |  | < .001 |  | < .001 |  | — |  |
| Knee extensors right max RTD [Nm/s] |  | 0.397 | *** | -0.050 |  | -0.070 |  | -0.014 |  | -0.076 |  | 0.348 | *** |
|  |  | < .001 |  | 0.378 |  | 0.215 |  | 0.802 |  | 0.178 |  | < .001 |  |
| Ankle plantar flexors max RTD [Nm/s] |  | 0.473 | *** | 2.871e-4 |  | 0.021 |  | -0.027 |  | 0.059 |  | 0.353 | *** |
|  |  | < .001 |  | 0.996 |  | 0.705 |  | 0.633 |  | 0.287 |  | < .001 |  |
| Hip extensors right max RTD [Nm/s] |  | 0.262 | *** | 0.123 | * | 0.108 |  | 0.118 | * | 0.045 |  | 0.337 | *** |
|  |  | < .001 |  | 0.028 |  | 0.053 |  | 0.034 |  | 0.421 |  | < .001 |  |

* p < .05, ** p < .01, *** p < .001
